# Cryobiopsy at the dawn of fibrosis: unlocking early diagnosis and therapeutic windows

**DOI:** 10.3389/fmed.2026.1821109

**Published:** 2026-08-12

**Authors:** Claudia Carducci, Naftali Kaminski, Aurélien Justet, Sara Tomassetti

**Affiliations:** 1 Department of Pulmonology, Lausanne University Hospital (CHUV), University of Lausanne, Lausanne, Switzerland; 2 Section of Pulmonary, Critical Care and Sleep Medicine, Yale School of Medicine, New Haven, CT, United States; 3 Service de Pneumologie, Centre de Competences de Maladies Pulmonaires Rares, Centre Hospitalier Universitaire de Caen University of Caen Normandie, CEA, Centre National de la Recherche Scientifique, Imagerie et Stratégies Thérapeutiques pour les Cancers et Tissus Cérébraux/CERVOxy Group, GIP CYCERON, Normandie University, Caen, France; 4 Unità Operativa Complessa Pneumologia Rimini Riccione, Rimini, Italy

**Keywords:** aberrant basaloid cell, early, fibrosis, ILA, ILD, interstitial lung abnormalities, interstitial lung disease, transbronchial lung cryobiopsy

## Abstract

Transbronchial lung cryobiopsy (TBLC) has rapidly established itself as a promising, less invasive alternative to surgical lung biopsy for the evaluation of interstitial lung diseases (ILDs). While its diagnostic accuracy and safety are increasingly recognized in clinically overt forms, its role within the more subtle spectrum of interstitial lung abnormalities (ILAs) and early ILDs remains insufficiently defined and therefore largely debated. In this expert narrative review, we explore the current state of evidence surrounding TBLC and its potential applicability in these emerging clinical entities, addressing technical variability, diagnostic performance, safety profile, and integration within multidisciplinary frameworks. We further examine the ethical and clinical tension arising from the use of invasive diagnostics to largely asymptomatic or minimally symptomatic individuals, where the balance between potential benefit and harm remains undefined. Beyond these pragmatic considerations, TBLC offers a unique vantage point into the onset of fibrotic remodeling: its broader application may unveil the earliest histopathologic and biological substrates of chronic and acute tissue injury in ILDs, including aberrant basaloid cells, potential indicators of maladaptive repair that may orchestrate the fibrotic cascade. By enabling recognition of usual interstitial pneumonia (UIP) at its inception, TBLC holds the promise not merely of refining risk stratification, but of opening new therapeutic horizons in which antifibrotic strategies may be deployed at a stage when disease is still modifiable. We contend that rigorously designed prospective studies are urgently needed to determine whether early tissue sampling with TBLC can transform ILAs from incidental findings into a window of therapeutic opportunity, thereby altering the otherwise progressive trajectory of fibrosing lung diseases.

## 1 Introduction

Transbronchial lung cryobiopsy is an innovative bronchoscopic technique for obtaining lung tissue in patients with suspected diffuse parenchymal lung disease. The procedure entails advancing a cryoprobe distally into the bronchus, where it is rapidly cooled to −75 °C with carbon dioxide (1) or −89 °C with nitrous oxide (2, 3), thereby freezing adjacent lung tissue that is subsequently retrieved en bloc with the probe (4). The cryosurgical equipment mechanism is based on the Joule-Thomson effect, whereby rapid gas expansion at the probe tip causes an abrupt temperature drop below 0 °C (5).

In recent years, TBLC has gained increasing traction in the evaluation of interstitial lung diseases, as it yields larger specimens with a higher proportion of alveolated tissue and fewer crush artifacts compared to conventional transbronchial forceps biopsies (2, 6). It has therefore emerged as a promising and potentially safer alternative to surgical lung biopsy, particularly for older patients (7), individuals with unclassifiable ILDs, including those at an early stage, and for individuals with interstitial lung abnormalities.

Despite a growing and substantial body of evidence, TBLC remains non-standardized, and its precise role within the diagnostic algorithm of ILAs and early ILDs is still undefined. Nevertheless, the acquisition of qualitatively and quantitatively adequate tissue remains pivotal in establishing a diagnosis when high-resolution computed tomography (HRCT), clinical assessment, and laboratory investigations prove inconclusive (8).

In this article, we aim to examine the unmet needs and challenges inherent in applying TBLC to ILAs and early ILDs, with the goal of clarifying its present utility and future potential.

## 2 Methods

This work was conceived as an expert narrative review, with the literature selected based on the authors’ expertise and the relevance of the studies to the topics discussed, which should be considered a limitation of the review. Foundational studies relevant to this topic are summarized in Table 1.

**TABLE 1:** Foundational studies informing the potential role of transbronchial lung cryobiopsy in interstitial lung abnormalities.

References	Aim	Design	Study groups	Key findings	Clinical relevance and limitations
Lachowicz et al. (9)	Assessment of cryobiopsy yield, safety, and procedural factors.	Systematic review and meta-analysis	70 studies included; pooled 6,183 participants.	• Diagnostic yield: 81% overall. • MDD increased diagnostic yield (82% vs. 76%). • Pneumothorax rate: 5%. • Moderate-to-severe bleeding: 12%.	Strength: largest synthesis of TBLC in ILDs evidence to date. Clinical relevance: supports TBLC as a high-yield and relatively safe diagnostic procedure for ILD. Limitation: considerable heterogeneity in procedural techniques and study protocols.
Zayed et al. (10)	To evaluate the diagnostic yield and adverse events of TBLC in patients with ILD.	Systematic review and meta-analysis	68 studies; 6,386 patients.	• Overall diagnostic yield: 82.3%. • Histopathologic yield: 72.5%. • Pneumothorax: 9.6%; chest tube required in 5.3%. • Severe bleeding: 1.9%. • Procedure-related mortality 0.9%.	Strength: comprehensive evaluation of diagnostic performance and safety. Clinical relevance: confirms TBLC as a less invasive alternative to surgical lung biopsy. Limitation: variability in patient selection and procedural approaches across studies.
Miller et al. (11)	Analysis of histopathology of ILAs in the context of lung nodule resections.	Retrospective study	424 patients undergoing lung nodule resection with CT scan within 3 months before surgery, and no history of ILD.	• ILA identified in 6% of patients, indeterminate ILA in 33%. • Patients with ILA were more likely to have subpleural fibrosis, fibroblastic foci and atypical adenomatous hyperplasia.	Strength: direct radiologic–pathologic correlation using surgical specimens. Clinical relevance: provides biological evidence linking ILA with early fibrotic lung disease. Limitation: retrospective design and absence of longitudinal clinical outcomes.
Chae et al. (12)	To evaluate radiologic and histologic correlations of ILA and their prognostic significance.	Retrospective diagnostic and prognostic study	Out of 268 patients undergoing surgical lung biopsy, 45 patients with incidentally detected ILA were selected.	• Subpleural fibrotic ILA associated with increased rate of progression. • Increased mortality risk compared with non-fibrotic ILA. • Median progression-free interval: 40 vs. 86 months.	Strength: combined imaging, pathology and outcome data. Clinical relevance: identifies fibrotic ILA as a high-risk phenotype with prognostic significance. Limitation: single-center retrospective design with potential selection bias.
Fukuda et al. (13)	To investigate the clinicopathological spectrum of Fleischner-defined ILA reclassified according to ATS 2025 criteria.	Retrospective study	30 patients undergoing surgical lung biopsy.	• UIP (43.3%), BIP (20%), and NSIP (20%) were the dominant patterns. • 97% met ATS 2025 criteria for subclinical ILD. • No significant survival difference between UIP and non-UIP groups.	Strength: histopathologic characterization using contemporary ATS criteria. Clinical relevance: supports reclassification of many ILA cases as subclinical ILD. Limitation: small sample size limits generalizability.
Hung et al. (14)	To determine the prevalence and characteristics of parenchymal findings in patients with lung nodules.	Retrospective study	397 patients with resected lung nodules and available presurgical CT scan.	• Parenchymal abnormalities identified in 25% of patients. • Fibrotic interstitial changes in 10%, and UIP in 1%.	Strength: large surgical cohort with histopathologic assessment. Clinical relevance: highlights the frequency of incidental interstitial abnormalities in routine thoracic practice. Limitation: limited follow-up and single-center retrospective design.

Although no studies have specifically evaluated TBLC in patients with ILA, the available evidence on TBLC in ILD and on the radiological, histopathological, and prognostic features of ILA provides the current framework for clinical decision-making and highlights the need for dedicated prospective investigations.

## 3 The Achilles’ heel of cryobiopsy in early disease

A central obstacle to the broader adoption of transbronchial lung cryobiopsy in early interstitial lung diseases and interstitial lung abnormalities is the absence of standardized protocols. This lack of uniformity directly influences diagnostic performance, introduces variability across centers, and continues to obscure the true value of the technique in these early or indeterminate settings. There are no guidelines for interventional pulmonologists regarding site selection, number of samples, probe diameter, and anesthesia protocols, which might impact the diagnostic yield, especially due to the patchy distribution of some pathological features in fibrotic diseases.

Additionally, inconsistencies in bleeding severity scales and in the interpretation of pneumothorax outcomes further complicate the assessment of safety and risk.

The technical parameters of cryobiopsy, such as gas pressure, freezing duration, number and size of specimens, and maximal specimen length, vary considerably across studies, with direct consequences for the diagnostic accuracy (15). For example, a positive correlation has been observed between freezing time and specimen size (5). Reported mean specimen areas range from 9 to 64.2 mm^2^, and an adequate cryobiopsy is generally considered to measure at least 5 mm × 5 mm (5, 8, 10, 16–18). For comparison, conventional transbronchial biopsies usually measure 0.1–0.3 cm, and surgical lung biopsies approximately 4 cm along the longest axis (19). Despite being markedly smaller than video-assisted thoracoscopic or open surgical biopsies, cryobiopsy specimens, particularly when multiple samples are obtained, often contain enough alveolated tissue to reveal histopathological patterns that are not discernible with conventional forceps biopsies (18).

Cryoprobe diameters are highly variable, a choice that can shape both specimen quality and procedural safety. The 2.4-mm cryoprobe has been shown to enhance diagnostic performance (9, 20, 21), but smaller probes can improve the adverse event profile. In a prospective randomized trial of 224 patients, no significant differences in specimen quality or diagnostic success were observed between the 1.9-mm and 1.1-mm probes; the smaller probe, however, reduced moderate bleeding but trended toward a higher risk of pneumothorax (22). The use of smaller probes slightly reduces diagnostic yield, to 80.4% and 79.5% for the 1.9-mm and 1.1-mm probes, respectively (22). Nevertheless, TBLC remains far superior to conventional transbronchial forceps biopsy, which achieves a diagnosis in around 48%–63.1% of cases across fibrotic and non-fibrotic ILDs (23, 24). The 1.7 mm appears to have a safety profile and diagnostic yield very similar to the 1.9 mm probe, although further studies are needed to confirm these findings (25–27).

Targeting multiple lung regions, particularly those exhibiting varying degrees of involvement or distinct radiological patterns, appears both logical and advantageous. In line with this, Ravaglia et al. (28) demonstrated that obtaining biopsies from different segments within the same lobe, rather than from a single segment, increased diagnostic yield from 69% to 96%. Conversely, limiting biopsies to areas showing exclusively fibrotic changes on HRCT is unlikely to reveal specific histopathological features and should therefore be avoided (5).

Cryobiopsy has emerged as a promising technique to increase diagnostic confidence in ILDs, with diagnostic yield and agreement in usual interstitial pneumonia potentially approaching those of the gold standard surgical lung biopsy (SLB), although the available evidence remains limited and of low quality (10, 29–35). SLB remains the benchmark, with reported diagnostic yields between 93.5% and 98% (36–38). When assessed within a multidisciplinary discussion (MDD), TBLC achieves diagnostic yields ranging from 78% to 87.9% (10, 17, 18, 23, 24, 36, 39–43), with a pooled estimate of 82.3% (95% CI 78.9–85.2) reported in a recent meta-analysis (10). Histopathological assessment alone yields slightly lower values, ranging from 72.5% (95% CI 67.7%–76.9%) to 80% (10, 17, 19, 36, 44, 45). In a more recent study by Liu et al., not included in the Zayed et al. meta-analysis, TBLC achieved a histological diagnostic yield of 88.9%, increasing to 97.6% after MDD (46). This could either be a statistical outlier due to specific study conditions or, conversely, a reflection of growing global expertise and technical refinements since the technique’s introduction.

In populations with unclassifiable ILD or low diagnostic confidence, TBLC has been shown to provide a definite or high-confidence diagnosis in 81% of cases (95% CI 79%–83%, I^2^ = 97%) (9). Although this estimate is derived from a population that differs from ILA and early ILD, it likely represents the closest conceptual and methodological comparator currently available. Nevertheless, direct data in ILA or early ILD populations are still lacking, and any extrapolation should therefore be interpreted with caution.

Given that ILAs remain an under characterized entity, there is increasing interest in developing and refining approaches that can enhance diagnostic yield while minimizing procedural risk, thereby allowing a broader and more appropriate spectrum of patients to be considered for biopsy.

Cone-beam computed tomography (CBCT) guidance appears to enhance safety compared with fluoroscopic guidance, while both approaches facilitate a multidisciplinary diagnosis (47–50). Also, the adoption of ancillary endoscopic tools such as confocal laser endomicroscopy or optical coherence tomography could refine diagnostic accuracy by allowing for more peripheral and precise biopsies with less complications (51–53). Combinations of chest CT angiography with radial ultrasound and fluoroscopic guidance have also been explored, enabling cryobiopsy in areas of maximal disease activity with minimal vascularization, thereby optimizing tissue acquisition (54, 55). Robotic-assisted platforms enable enhanced distal airway access through articulating catheters and computer-assisted navigation, improving stability and reach within subsegmental bronchi (56, 57). Electromagnetic navigation bronchoscopy provides three-dimensional CT-based pathway guidance to target subtle parenchymal abnormalities and superior accuracy compared to traditional fluoroscopy (58–60), while virtual bronchoscopic navigation systems offer CT-derived airway reconstruction to support pre-procedural planning and intraprocedural guidance through computational modeling of the bronchial tree (61). These navigation strategies are increasingly used within integrated interventional workflows and are often combined with advanced imaging modalities, particularly cone-beam CT and radial endobronchial ultrasound, to improve lesion localization, confirm tool-in-lesion positioning, and optimize sampling accuracy in real time (62, 63). Within the bronchoscopy session itself, rapid on-site evaluation (ROSE) offers the potential to further enhance outcomes. Ongoing trials are investigating the diagnostic performance of TBLC with ROSE compared with conventional transbronchial lung biopsy (TBLB), aiming to clarify how biopsy timing, freezing duration, and specimen size influence both diagnostic accuracy and complication rates (64). In spite of that, ROSE is routinely used for cytology in endoscopic procedures but is not available for histology due to processing delays; Higher Harmonic Generation (HHG) microscopy may bridge this gap by enabling near real-time evaluation of unprocessed biopsy samples in ILD TBLC (65).

Beyond safety, these integrated approaches may further improve the technical feasibility of TBLC through more reliable access to peripheral and radiologically subtle targets, particularly in ILAs. General anesthesia has been associated with a higher diagnostic yield, albeit at the cost of an increased risk of pneumothorax. This may reflect the greater control over cough and patient movement for the first, and the use of positive-pressure ventilation for the latter (9).

Several studies have demonstrated that TBLC, when performed at experienced bronchoscopy centers using a cryoprobe under conscious sedation rather than general anesthesia, is safe, well tolerated, and achieves a high diagnostic yield of over 85% in multidisciplinary evaluation (16, 66, 67). Nonetheless, larger trials directly comparing conscious and general sedation remain warranted. Diagnostic yield appears higher in patients with preserved lung function, particularly those with higher Diffusing capacity of the Lungs for Carbon Monoxide (DLCO) and Forced Vital Capacity (FVC) (9, 10), potentially because more tissue can be obtained in individuals who tolerate the procedure better, or because the presence of less dense fibrosis and more active disease facilitates definitive histopathological diagnosis (10). These observations underscore the importance of lung function as a key determinant of TBLC outcomes, suggesting that performing cryobiopsy earlier in the disease course, when DLCO and FVC are relatively preserved, may enhance both safety and diagnostic accuracy (9, 17).

Safety remains a central concern in the widespread adoption of TBLC, and overall complication rates are reassuringly low. Pneumothorax occurs in approximately 5% of cases (9, 36), with higher rates reported when using a 2.4-mm cryoprobe, routine post-procedure imaging, sampling across multiple lobes, reduced DLCO, general anesthesia (9), and presence of visceral pleura in the sample (68). Conversely, lower pneumothorax rates are observed in high-volume centers performing ≥70 TBLC annually (36), and when fluoroscopic guidance is employed (39).

Lung ultrasound performed immediately after TBLC provides valuable clinical guidance, enabling early detection of pneumothorax requiring intervention before extubation, and permitting real-time monitoring of pneumothorax size in the operating room. Nonetheless, supplementary chest radiography prior to discharge remains essential, as most pneumothoraxes develop later in the post-procedure period (69). Bleeding complications are generally classified according to the British Thoracic Society (BTS) (70) or Nashville guidelines (71) (Table 2).

**TABLE 2:** British thoracic society and Nashville guidelines bleeding classification.

Nashville grade	Nashville description	BTS severity level	BTS description
Grade 1	Requiring <1 min of suctioning or wedging of the bronchoscope resulting in spontaneous cessation of bleeding.	No bleeding	Traces of blood with no need for continuous suctioning. Bleeding stops spontaneously.
Grade 2	Suctioning >1 min or need for rewedging, or instillation of cold saline, vasoactive substances, or thrombogenic agents.	Mild bleeding	Continued suctioning of blood from the airways. Bleeding stops spontaneously.
Grade 3	Selective intubation with endotracheal tube or balloon/bronchial blocker for <20 min, or premature interruption of the procedure.	Moderate bleeding	Intubation of the biopsied segment with the bronchoscope in wedge position. Use of adrenaline or cold saline to stop bleeding.
Grade 4	Persistent selective intubation >20 min, or new ICU admission, packed RBC transfusion, bronchial artery embolization, or need for resuscitation.	Severe bleeding	Placement of bronchus blocker or catheter, applying fibrin sealant. Resuscitation, blood transfusion, ICU admission, or death.

Moderate bleeding occurs in approximately 11.7% of cases, while severe bleeding is rare (10), ranging from 1.6% (36) to 1.9% (9). However, these estimates should be interpreted cautiously, as they are derived from heterogeneous studies using different bleeding classification systems (including Nashville and BTS scales, Table 2), which limit direct comparability across reported incidence rates.

Risk increases with sampling from multiple segments (10), with the use of a 2.4 mm cryoprobe, and depending on the bleeding score applied (9). Routine use of endobronchial balloon blockers is widely recommended (5, 8, 72), though their statistical impact appears modest: they may attenuate moderate bleeding (73), perhaps severe bleeding in some series (39, 74), yet consistently without improving diagnostic yield (73, 74).

Mortality from TBLC is strikingly low; 0.9% versus 2.4% with SLB (10) most often from respiratory failure or acute ILD exacerbation (10, 39, 75). Fatal bleeding has not been reported (10). By contrast, SLB mortality is compounded by pneumonia, empyema, and acute exacerbations, potentially precipitated by surgical factors such as alveolar collapse during VATS, barotrauma with single-lung ventilation, or oxygen toxicity (8, 36).

Transbronchial lung cryobiopsy has shown safety even in patients considered at risk owing to advanced age, obesity, impaired lung function, or cardiac comorbidities (76). Predictors of 30–90 days hospitalization include mMRC ≥2, FVC <50%, and Charlson Comorbidity Index ≥2 (77).

Yet, the risk of acute exacerbation, especially with higher biopsy numbers, remains an important consideration. Clearly, not all cryobiopsies carry equal risk: procedures in hospitalized patients are associated with higher rates of pneumothorax, air leak, ICU transfer, and 30-days mortality (78).

Optimal safety and yield are achieved in high-volume centers, underscoring the value of structured training (72, 79). In fact, TBLC is now endorsed as the preferred histological approach for suspected fibrosing ILDs when performed in expert centers (79–81). SLB remains appropriate after a non-informative TBLC (79), even if the utility of SLB after a non-diagnostic TBLC appears limited, with reclassification in only 23% of cases (10, 82).

While SLB has traditionally been avoided in HRCT-defined UIP, the reduced morbidity of TBLC broadens the role of histology. As a matter of fact, it can be used in ILDs with uncertain exposures or occult connective tissue disease, where biopsy may reveal granulomas, organizing pneumonia, or lymphoid aggregates directing diagnosis (8).

In pediatric ILDs, TBLC has achieved diagnostic accuracies comparable to SLB, though validation in larger, standardized studies is required (83).

To synthesize the key features of each technique and facilitate an immediate, side-by-side comparison, a comparative overview of transbronchial lung cryobiopsy and surgical lung biopsy is presented in Table 3, delineating their respective strengths and limitations across the spectrum from interstitial lung abnormalities to established interstitial lung disease.

**TABLE 3:** Comparative overview of transbronchial lung cryobiopsy and surgical lung biopsy in the diagnostic evaluation of interstitial lung diseases, derived from the literature appraised in this narrative review.

	Transbronchial lung cryobiopsy	Surgical lung biopsy
Procedure type	Less invasive bronchoscopic technique	More invasive surgical procedure, typically Video-assisted thoracic surgery (VATS)
Anesthesia	Conscious sedation or general anesthesia	General anesthesia with single-lung ventilation
Specimen size	∼5–64 mm^2^ (multiple samples)	∼3–4 cm (larger, en bloc specimen)
Tissue quality	Good alveolated tissue, minimal crush artifact; limited architectural context	Optimal architectural preservation; reference standard for pattern recognition
Spatial sampling limitation	Present; may miss disease heterogeneity due to localized sampling	Minimal; allows comprehensive architectural assessment
Sampling strategy	Multiple samples (≥2–5), often from ≥2 segments/lobes to improve yield	Typically 1–2 lobes selected based on radiologic guidance
Diagnostic yield (histology)	∼68%–77%	∼95%
Diagnostic yield (MDD)	∼79%–85%	∼93% (with fewer studies available than for SLB histological yield)
Agreement with SLB	High concordance for UIP, especially within MDD (∼70%–80%); lower for non-UIP patterns; overall low-quality evidence.	Reference standard
Ability to detect UIP pattern	Good, but may be limited by sampling variability	Optimal detection due to full architectural assessment
Complication rate (overall)	Low	Moderate
Pneumothorax	∼5%–10%; higher with larger probes and multilobar sampling	Not a complication per se (pleural access intentional; chest tube routinely placed)
Bleeding	Moderate ∼10%–12%; severe ∼1%–2%	Rare intraoperative bleeding; postoperative complications possible
Acute exacerbation risk	Low (∼1%–2%)	Higher (∼2%–5%)
Mortality	∼0.5%–1%	∼2%–2.5%
Hospital stay	Short (often outpatient or ≤1–2 days)	Longer (typically several days)
Time to diagnosis	Shorter; integrated within bronchoscopic workflow	Longer; requires surgical scheduling and recovery
Feasibility in frail patients	Generally feasible, including elderly and comorbid patients	Often contraindicated in high-risk patients
Cost and resource use	Lower; endoscopy suite-based	Higher; requires operating room and surgical team
Standardization	Limited; variability across centers	Well standardized
Center dependency	High; strongly influenced by operator expertise and center volume	Moderate; more reproducible across centers
Learning curve	Significant; operator-dependent	Established surgical expertise
Role in diagnostic algorithm	First-line histologic modality in many centers when tissue is required, particularly in expert settings	Reserved for selected cases or after non-diagnostic TBLC
Utility in early ILD/ILA	Non recommend for lack of studies, although promising	Limited due to invasiveness
Suitability for molecular analyses	Good; compatible with transcriptomics and other omics approaches	Excellent; larger tissue volume allows extensive analyses

Further research to optimize patient outcomes and procedural safety are needed (75), especially when thinking about enlarging the cohort of candidates to ILAs and early ILDs carriers. Importantly, the current safety estimates we described derive from cohorts with established disease and may therefore overestimate procedural risk in earlier stages. On the other hand, ILA are characterized by limited radiologic involvement, preserved lung architecture, and near-normal pulmonary function (84, 85). The relative absence of advanced fibrotic remodeling, especially subpleural honeycombing and marked architectural distortion, likely reduces susceptibility to pleural injury, with a consequent lower risk of pneumothorax. In parallel, more preserved parenchymal and vascular integrity may favor more controlled bleeding compared to the fragile, traction-altered tissue typical of advanced ILD. Although targeting subtle abnormalities may require more peripheral sampling, this technical aspect is unlikely to offset the intrinsically lower structural vulnerability of the lung in ILA. Accordingly, complication rates reported in overt ILD may represent a worst-case scenario, rather than a benchmark applicable across the disease spectrum. However, we must also consider that in individuals who are often asymptomatic and functionally preserved, even modest reductions in procedural risk could meaningfully shift the risk-benefit balance.

Finally, defining the safety profile of TBLC in well-characterized ILA populations represents a key priority for future prospective studies, with direct implications for patient selection and the design of early-disease trials.

## 4 Stratifying risk, anticipating progression from ILA to early UIP: Can TBLC illuminate the path?

The natural history of ILA and early ILD remains poorly defined, particularly regarding how early tissue sampling might alter the trajectory of the nascent disease. While transbronchial lung cryobiopsy carries a risk profile more favorable than surgical lung biopsy, complications, although rare, still exist. Understood that a scenario where every incidental abnormality is subjected to biopsy is dystopian, the pressing mandate is assessing the risk of progression to a clinically open ILD (86), and stratifying ILA and early ILDs carriers according to a rational and evidence-based hierarchy of risk.

To date, the vast corpus of TBLC data has been derived from clinically overt ILD populations; limited evidence in early ILD, and screening-detected or incidental ILA exists.

What is known, however, is that the trajectory of ILAs tends toward progression rather than stability, with nearly half of carriers exhibiting worsening over time (87). Reported progression rates vary widely between 20% and 80% (88–92), and evolution toward a UIP pattern has been shown in 17.3% of cases in less than 12 years (91).

Several factors consistently associate with increased risk, including advanced age, smoking history, connective tissue disease, environmental exposures, and familial predisposition. Among the genetic contributors, the MUC5B promoter variant and telomere disorders further amplify susceptibility. Even physiologic markers, when merely borderline in FVC, TLC, or DLCO, may suggest early impairment (85). In fact, in ILAs and early ILDs, pulmonary function can appear normal, even when subtle alterations are already present, especially in the presence of emphysema (93). Notably, individuals with Preserved Ratio Impaired Spirometry (PRISm; FEV_1_ < 80% predicted with FEV_1_/FVC > 0.7) show significantly higher odds of ILAs compared with those with normal spirometry (94). This highlights how conventional thresholds may overlook subtle functional decline, reinforcing the need for refined stratification strategies.

It is not only early ILDs but also ILAs that portend an increased risk of mortality. This association has been widely established, although the reported magnitude varies remarkably across cohorts from modest relative risks of 0.92 to noteworthy values as 12.43 (89, 94–100), with a pooled estimate of 1.66 (85).

Furthermore, certain features within the ILA patterns are strikingly more impactful; fibrotic ILA (90), especially when accompanied by traction bronchiectasis (90, 101–105) or honeycombing (85), newly identified ILAs, as opposed to persistent interstitial changes which may reflect residual scarring or stable abnormalities (106), convey markedly elevated risks of adverse outcomes. In fact, fibrotic ILA, as a subgroup, is consistently associated with the greatest risk of progression and mortality (85, 89, 94–100), suggesting that individuals exhibiting these radiologic hallmarks may warrant more frequent radiological surveillance than the conventional 3-years interval (91). Revealingly, in individuals at risk for familial pulmonary fibrosis, even the subtlest interstitial abnormalities occupying less than 5% of a lung zone, may already herald subclinical disease (107).

Complementary tools should be employed whenever possible to more accurately stratify patients at high risk of progression and to better characterize them, guiding the subsequent decision between pursuing a more aggressive TBLC approach or maintaining a conservative strategy.

Among serum biomarkers, MMP7 is a marker of epithelial transformation associated with increased odds of ILA (108), while KL-6 (Krebs von den Lungen-6) is increased in injured or regenerating epithelial cells, correlates with radiological progression, and may help identify ILA carriers at higher risk of fibrotic evolution (109). Along KL-6, SP-D (Surfactant Protein D) is also significantly increased in ILA, especially the fibrotic subtype (110, 111) and the familial cases (112).

A more robust link (COPDGene, AGES-Reykjavik, and the Framingham Heart Study) has been established between telomere length and ILA (113), which could be tested especially in individuals with familiarity for ILDs or other signs related to the syndrome of shorter telomeres, even in other organs besides the lungs.

Lastly, the integration of quantitative CT analysis (e.g., High Attenuation Areas) provides an objective measure of early interstitial changes, associated with increase of extracellular matrix remodeling biomarkers, offering a non-invasive longitudinal alternative to monitor stability (114).

By framing TBLC within this multimodal approach, clinicians can better differentiate between “senescence-related” ILA (likely stable) and “early-stage ILD” (likely progressive), thereby reducing the risk of overdiagnosis and unnecessary invasive procedures.

Altogether, the integration of these clinical, radiological, and molecular features identifies a higher-risk profile for disease progression, which may support the decision to pursue lung biopsy for diagnostic clarification. However, in cases where the multidisciplinary evaluation already yields a high diagnostic confidence (>70%), a biopsy may be reasonably deferred, favoring a non-invasive management approach (115).

## 5 Ethics at the interface of early diagnosis and invasive sampling

Prior to embarking on TBLC, one must confront the ethical tension inherent in subjecting asymptomatic individuals to an invasive biopsy, especially in light of a therapeutic risk-benefit equilibrium that remains, to date, elusive and insufficiently defined (84, 100, 116).

In the context of ILAs or of minimally symptomatic early ILDs, decisions regarding tissue sampling must be patient-centered, ensuring that individuals are fully informed about the potential benefits, limitations, and risks of the procedure, as well as the potential physical and psychological burden imposed (106). Moreover, antifibrotic agents are associated with frequent adverse events, including gastrointestinal disturbances, like nausea, diarrhea and weight loss, hepatotoxicity, photosensitization, headache, and fatigue, which can necessitate dose adjustments or discontinuation (117–121). Depending on the health care system, very high annual drug costs for antifibrotics might further compound the burden. Immunosuppressive therapies, typically reserved for inflammatory or autoimmune ILDs, carry additional risks such as infections, cytopenias, metabolic disturbances, and organ toxicity, particularly in older or comorbid individuals (122).

Crucially, patients should be informed that robust evidence supporting the benefits of early therapeutic intervention remains limited. Although the rationale for initiating treatment at an earlier stage is compelling, offering the potential to prolong survival and preserve quality of life in diseases still lacking truly disease-reversing therapies, this approach remains, at present, insufficiently supported by definitive clinical evidence (123).

## 6 Informative power and interpretative challenges of histopathology

The critical role of tissue in distinguishing ILA and early ILD from established ILD has been emphasized in the recently published ILA guidelines (85), where histopathology is recognized as a key diagnostic criterion for confirming a definitive diagnosis. At the same time, the guidelines advise against routine lung biopsy, highlighting the inherent tension between the acknowledged value of tissue understanding and the practical limitations of its acquisition.

The limited data we do have provides compelling, albeit fragmented, evidence of significant histopathological changes in patients with ILAs. A cohort of individuals with radiological ILAs, who underwent lung nodule resection, were found to have a higher prevalence of subpleural fibrosis, fibroblastic foci, and atypical adenomatous hyperplasia compared to those without ILAs (11). In a retrospective study (12) involving patients with radiological ILA undergoing surgical biopsy, 69% of subpleural fibrotic ILA showed definite or probable UIP patterns, while 11% of subpleural non-fibrotic ILA showed definite or probable UIP patterns (12). Moreover, a prospective study on explanted lungs revealed that while areas of reticulation on CT scans generally correlated with greater fibrosis, varying degrees of fibrosis were also present in regions that appeared normal on CT. This underscores the limitations of imaging alone and reinforces the need for a deeper, tissue-level analysis to fully grasp the extent of the disease (124). Additionally, when imaging or clinical features appear NSIP-like, TBLC could clarify the true histopathological UIP substrate (125), which is linked with poorer survival (126, 127), including in early ILD and ILAs where histological UIP may be present despite non-specific radiological findings.

This potential for deeper diagnostic resolution, however, coexists with a non-negligible risk of overdiagnosis and histologic misclassification, a key consideration in ILAs and early ILDs, as limited or focal abnormalities captured by small biopsy samples may be overinterpreted as indicative of fibrosing patterns, even when they do not reflect a true, spatially and temporally established fibrosing interstitial lung disease (85, 128). This inherent uncertainty highlights a key challenge: not only whether tissue sampling should be performed, but also when, how, and in whom it can be most appropriately pursued. Specifically, careful patient selection is critical to optimize diagnostic yield, maximize clinically meaningful diagnoses, and minimize overdiagnosis of benign lesions. In fact, in studies where surgical lung biopsies were performed specifically for ILA evaluation, Chae et al. reported probable or definite UIP in 58% of all ILA cases and in 69% of radiologically fibrotic ILAs (12), while Fukuda et al. identified dominant or co-existing UIP patterns in approximately 60% of patients with ILA (13). In contrast, studies based on incidental tissue specimens obtained during surgery for other indications showed substantially lower rates of UIP, with Miller et al. reporting UIP in only 8% of cases (11), and Hung et al. identifying UIP in 25% (14). Although these studies are inherently limited by their retrospective design and, in some cases, small sample sizes, they importantly highlight that the clinical impact of tissue sampling in ILAs is closely linked to the rigor of upstream decision-making.

Lastly, even when the upstream decision is appropriate and the procedure is performed optimally, the downstream interpretation may still prove challenging. Notably, in cases of suspected early ILD or ILA, there are currently no specific guidelines to standardize how pathologists should report their findings. What we know is that interpreting cryobiopsy samples involves a learning curve, and prior experience with surgical lung biopsies in diffuse lung disease is invaluable. Ideally, a standardized report should detail the biopsy site, tissue dimensions, and histological pattern (19, 128). Given that cryobiopsies are smaller and may provide less architectural information than surgical specimens, pathologists should also qualify their impressions with “low” or “high” confidence (18). Zaizen et al. recommend that standard hematoxylin and eosin staining should always be complemented by elastic fiber stains, such as Elastica van Gieson, as they significantly improve the assessment of fibrosis quality and alveolar architecture in small biopsy samples (129). They also recommend that even minimal fibrosis should not lead to exclusion of fibrotic ILD or to an alternative diagnosis based solely on IPF guidelines. A broad multidisciplinary evaluation is advised, keeping IPF, fibrotic hypersensitivity pneumonitis, and CTD-associated UIP pattern within the diagnostic considerations, also because TBLC generally does not capture subpleural lesions, making the assessment of subpleural fibrosis, one of the key features of UIP in SLB, difficult (129). Ultimately, tissue alone is not enough, its value emerges only within a broader clinical framework, such as the multidisciplinary discussion.

## 7 Orchestrating expertise: the multidisciplinary approach

In the intricate landscape of ILAs and early ILDs, the multidisciplinary discussion emerges as an indispensable arbiter, orchestrating the delicate tasks of classification, risk stratification, and management. At its core, the team comprises chest physicians, radiologists, and pathologists, yet in larger referral centers, participants from rheumatology, immunology, transplant medicine, palliative care, and clinical nursing often contribute, enriching the deliberative process (130).

The MDD has been consistently shown to enhance diagnostic concordance, particularly in unclassifiable cases such as ILAs and early ILDs, where the dynamic interplay of expertise fosters heightened diagnostic confidence and frequently converts previously indeterminate cases into specific diagnoses (44, 131, 132).

In the context of challenging histopathologic interpretation, the MDD ensures that biopsy findings are thoughtfully integrated with clinical and radiologic data, yielding a comprehensive and coherent assessment (18).

In the COLDICE study, histopathological agreement between TBLC and SLB specimens was 70.8%, which increased to 76.9% diagnostic agreement following MDD, underscoring its cardinal role in consolidating diagnostic consensus and confidence (44). Given the absence of universally accepted reporting guidelines among pathologists, the joint evaluation of histological features within the clinical-radiologic milieu is essential (133).

A discussion prior to the biopsy should be encouraged, as it may promote the selection of appropriate candidates, the exclusion of the inappropriate ones, and the careful balancing of procedural risks and benefits (130). Such discourse can also guide the identification of optimal biopsy sites, weighing factors such as proximity to the pleura against the need to target radiologic abnormalities. Moreover, by increasing the diagnostic accuracy (9), the MDD can reduce the invasiveness of a second procedure for the patients and deliver the highest standard care.

## 8 Complete lack of tissue biology knowledge in ILA carriers

While multidisciplinary assessment remains central, ILAs are still largely defined by radiological and clinical surrogates, leaving a critical gap in our understanding of the biological processes driving disease initiation and progression. At this juncture, the prevailing paradigm of observational medicine is insufficient to fully capture the latent biological risks carried by these individuals. In this context, obtaining tissue samples becomes pivotal to bridging this knowledge gap and may extend the role of biopsy beyond conventional histopathological description toward a more integrated framework encompassing characterization of molecular mechanisms driving pulmonary fibrosis. Indeed, the ability of TBLC to provide relatively large samples with well-preserved tissue architecture makes it particularly suitable not only for standard histological evaluation, but also for a broad range of downstream molecular analyses, including spatial and conventional transcriptomic profiling, next-generation sequencing (NGS), epigenetic characterization, and proteomic interrogation, thereby enabling a more comprehensive, multi-layered understanding of disease biology (134). We know that the composition of epithelial cells in fibrotic lung tissue, regardless of the cause, is characterized by the presence of aberrant basaloid cells, an abnormal cell phenotype with pro-fibrotic and senescent features, localized to the surface of fibrotic foci (135). These cells are hallmark of diseases ranging from IPF (135, 136), systemic sclerosis (137), and COPD (135) to COVID-19-induced fibrosis (138, 139), extending their relevance from chronic to acute etiologies of pulmonary fibrosis.

Since maladaptive epithelial plasticity could be the key mechanistic event in the shift of alveolar epithelial cells from injured witnesses to protagonist drivers of disease (140, 141), identifying this mechanism in the earliest stages of ILD and in ILAs is crucial.

An example of this biology-based approach is provided by the Envisia Genomic Classifier, a machine learning-based assay that analyzes gene expression from RNA extracted from transbronchial biopsy samples to identify a molecular signature consistent with UIP. The classifier is based on the expression of 190 genes, capturing pathways related to cell adhesion, migration, and profibrotic signaling that are enriched in UIP, in contrast to immune-related signatures seen in non-UIP patterns (142). Trained against surgical lung biopsy-proven cases and prospectively validated in the multicentre BRAVE study, with an 86% concordance with MDT diagnoses incorporating histopathology (143), Envisia provides a robust molecular surrogate of UIP. When applied in the context of TBLC, it has been shown to predict worse ILD progression over sustained follow-up, enabling early risk stratification without the need for surgical biopsy (144–146). Confirming the presence of these cellular phenotypes in ILAs would shift the paradigm from an observational approach to a targeted, precision medicine model. It could pave the way for a new therapeutic strategy focused on restoring normal epithelial differentiation, potentially offering a branch of regenerative medicine aimed at blocking fibrosis at its inception.

## 9 Operational framework for patient selection and trial design

Having explored the strengths, limitations, and unresolved challenges of tissue sampling, the focus now shifts toward its translation into an operational framework for clinical practice. To address this need, we propose a preliminary risk-stratification table integrating radiologic, functional, genetic, and clinical parameters (Table 4). Based on these parameters, patients are categorized into high, intermediate, or low-risk profiles to guide consideration of TBLC in expert centers, enabling a more actionable and standardized approach to ILA and early ILD patient selection.

**TABLE 4:** Decision pathway for ILA and early ILD patient selection for TBLC.

Domain	Parameter	Operational definition and threshold	Interpretation
Radiology (HRCT)	Fibrotic features	Presence of traction bronchiectasis and/or honeycombing	Strongest predictor of progression; key driver for TBLC consideration
	Extent of abnormalities (quantitative CT)	>10% lung involvement (147)	Higher extent increases likelihood of clinically relevant disease
Pulmonary function	FVC	<80% predicted or ≥80% predicted but disproportionately reduced vs. expected	Inyoung/fit individuals, “normal” values may mask early decline; interpret relative to expected peak (∼100%–120%)
	DLCO	<80% predicted or ≥80% predicted but disproportionately reduced vs. expected	Sensitive marker of early disease, even when FVC is preserved
	Longitudinal change	≥5% absolute decline in FVC or ≥10% relative decline in DLCO in 1 year	Suggests progression even if baseline values are normal
Genetic risk	MUC5B promoter variant	Presence of risk allele (if tested)	Associated with fibrotic progression
	Telomere-related abnormalities	Short telomeres (<10th percentile for age) or pathogenic variants (if tested)	Suggests more aggressive disease course
Clinical context	Connective tissue disease (CTD)	Established CTD diagnosis or suggestive autoimmune features	Higher pre-test probability of ILD and progression
	Familial ILD	≥1 first-degree relative with ILD	Asdefined in ERS statement (148); suggests genetic susceptibility
	Smoking status	Current/former heavy smoker	Higher pre-test probability
	Environmental exposure	Relevant fibrogenic exposures (e.g., occupational, high pollution exposure, hypersensitivity)	Higher pre-test probability
		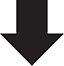	
		**Risk stratification**	**Proposed management strategy**
High-risk profile		Fibrotic ILA + ≥1 additional risk factor above	TBLC may be considered in expert centers
Intermediate-risk profile		Limited fibrosis or isolated risk factors	Individualized MDD based decision
Low-risk profile		Non-fibrotic ILA, no risk factors, stable function	Prefer surveillance

**FIGURE 1:**
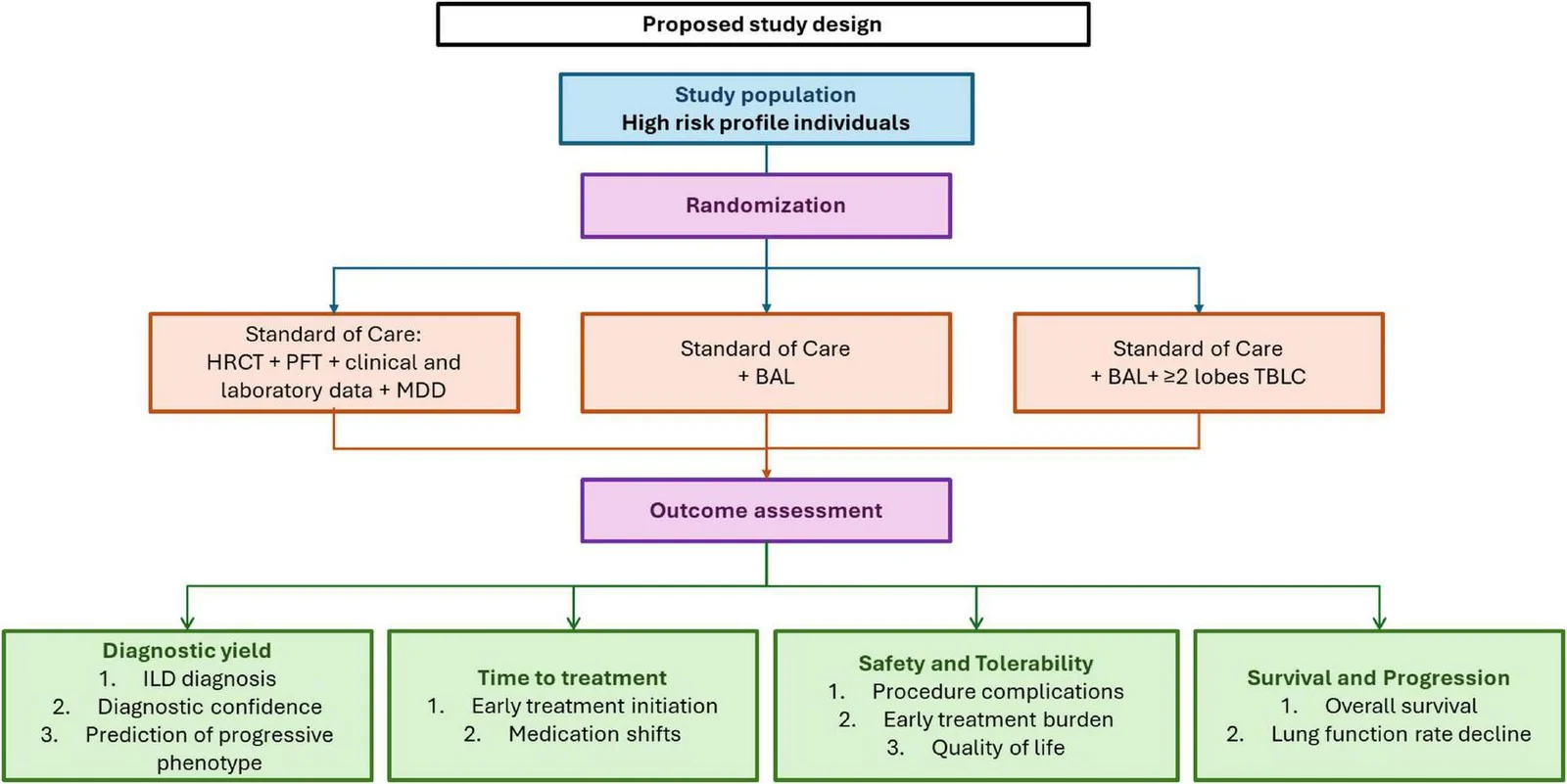
Proposed multicenter, prospective, randomized controlled study in patients with a high-risk profile. MDD, multidisciplinary discussion; PFTs, pulmonary function tests; BAL, bronchoalveolar lavage. Courtesy of the archives of the Department of Pathology, Careggi University Hospital, University of Florence, Italy.

As per this stratification, patients with a high-risk profile may be considered for TBLC in expert centers within a carefully selected clinical context. Building on this model, we outline a conceptual prospective multicenter study design to evaluate TBLC in ILA and early ILD cohorts, focusing on safety, diagnostic yield, and its potential impact on prognostication, management, and clinical outcomes (Figure 1).

To address ethical concerns, cryobiopsy would be restricted to those carefully selected patients, applying strict inclusion and exclusion criteria, in terms of pulmonary function tests (PFTs) and comorbidities, to minimize procedural risk. Procedures should be performed exclusively in high-volume expert centers by experienced operators, following standardized safety protocols and predefined stopping rules, alongside comprehensive informed consent and a clear risk-benefit justification.

Within this framework, participants would be randomized into three arms: (1) standard of care, including HRCT, PFTs, clinical and laboratory evaluation, and multidisciplinary discussion; (2) standard of care plus bronchoalveolar lavage; and (3) standard of care plus BAL and transbronchial lung cryobiopsy [sampling at ≥2 lobes (149, 150)].

Outcomes would be centrally adjudicated and would include diagnostic yield, time to treatment initiation, treatment modification rates, safety and tolerability, and clinically meaningful outcomes. The study is envisioned as a multicenter international collaboration to ensure adequate recruitment, with an estimated sample size of approximately 200 patients per arm, based on an anticipated 20% increase in diagnostic yield; final numbers may be refined according to feasibility and interim assumptions.

Diagnostic definitions would follow established frameworks, including ILD diagnosis criteria according to Ryerson CJ et al. (151), and progression according to Raghu G et al. (80). Multidisciplinary discussion would serve as the reference diagnostic standard. Treatment strategies would incorporate approved agents such as Nintedanib, Pirfenidone, Nerandomilast, Treprostinil, and combination approaches according to FDA approvals (117–121). Finally, parallel biobanking of TBLC samples would enable translational analyses, supporting biomarker discovery and future therapeutic innovation.

## 10 Discussion

The renewed interest in transbronchial lung cryobiopsy reflects an increasing effort to balance diagnostic accuracy with early detection of fibrotic lung disease. In the setting of interstitial lung abnormalities and early interstitial lung diseases, this tension becomes particularly evident: while current guidelines appropriately prioritize safety and generally discourage invasive sampling in asymptomatic or minimally symptomatic individuals, such a conservative stance may inadvertently perpetuate an observational paradigm that leaves unanswered the central biological questions underpinning disease initiation and early progression.

Emerging evidence suggests that TBLC may provide a unique histopathological window into the earliest manifestations of fibrotic remodeling. Even in cases characterized by subtle or radiologically indeterminate ILAs, biopsy specimens have revealed subpleural fibrosis, fibroblastic foci, and, in selected cases, patterns consistent with UIP. These observations challenge the implicit assumption that ILAs invariably represent benign or non-progressive entities and instead support the concept of a biologically heterogeneous continuum, in which fibrotic programming may already be operative long before clinical or functional decline becomes apparent.

At a deeper level, TBLC also offers a translational bridge between morphology and mechanism. The identification of aberrant basaloid cells as a recurrent feature of maladaptive epithelial repair across fibrotic lung diseases has reshaped our understanding of disease biology. The possibility that such cellular phenotypes may already be present in the ILA stage raises the provocative hypothesis that ILAs are not merely radiologic curiosities, but early biological expressions of epithelial-mesenchymal dysfunction. In this framework, histological sampling may transcend descriptive pathology and become a tool for mechanistic stratification, potentially enabling the identification of individuals in whom fibrogenesis is already silently active.

However, these conceptual advances must be interpreted within the current boundaries of clinical practice. At present, no antifibrotic therapy is approved for ILAs, and interventional evidence remains confined to established or progressive fibrotic ILDs. Ongoing therapeutic trials, including those evaluating Nintedanib, Pirfenidone, and newer agents such as Nerandomilast and Treprostinil, have not yet extended their inclusion criteria to asymptomatic or screen-detected ILA populations. In parallel, the absence of validated biomarker-driven algorithms to guide early therapeutic escalation further limits the immediate clinical translatability of a biopsy-informed precision approach.

Within this context, the role of TBLC in ILAs and early ILD should be viewed not as a routine diagnostic step, but as a hypothesis-driven instrument to be deployed in carefully selected, high-risk individuals. The critical challenge ahead lies in defining where along the spectrum of interstitial abnormalities tissue sampling becomes not only justifiable, but informative enough to meaningfully alter risk stratification and, potentially, clinical trajectory. This necessitates prospective, multicenter studies explicitly designed to evaluate safety, diagnostic yield, and, most importantly, whether histopathological information derived from TBLC can influence prognostic assessment and therapeutic decision-making.

Equally essential is the integrative function of the multidisciplinary team. In early disease states, where radiologic findings are subtle and histologic changes may be focal or ambiguous, the convergence of clinical, radiological, and pathological expertise remains indispensable. The MDD acts as a safeguard against both underdiagnosis and overinterpretation, ensuring that tissue findings are contextualized within a broader clinical and imaging framework, thereby maintaining diagnostic rigor while avoiding premature therapeutic escalation.

Taken together, TBLC has already established itself as a cornerstone in the diagnostic work-up of fibrotic ILDs. Its most transformative potential, however, may reside upstream in the disease course, where structural and molecular changes begin to diverge from radiological subtlety. In this regard, ILAs and early ILD should be conceptualized not as static entities, but as dynamic points along a continuum of fibrotic evolution.

**FIGURE 2:**
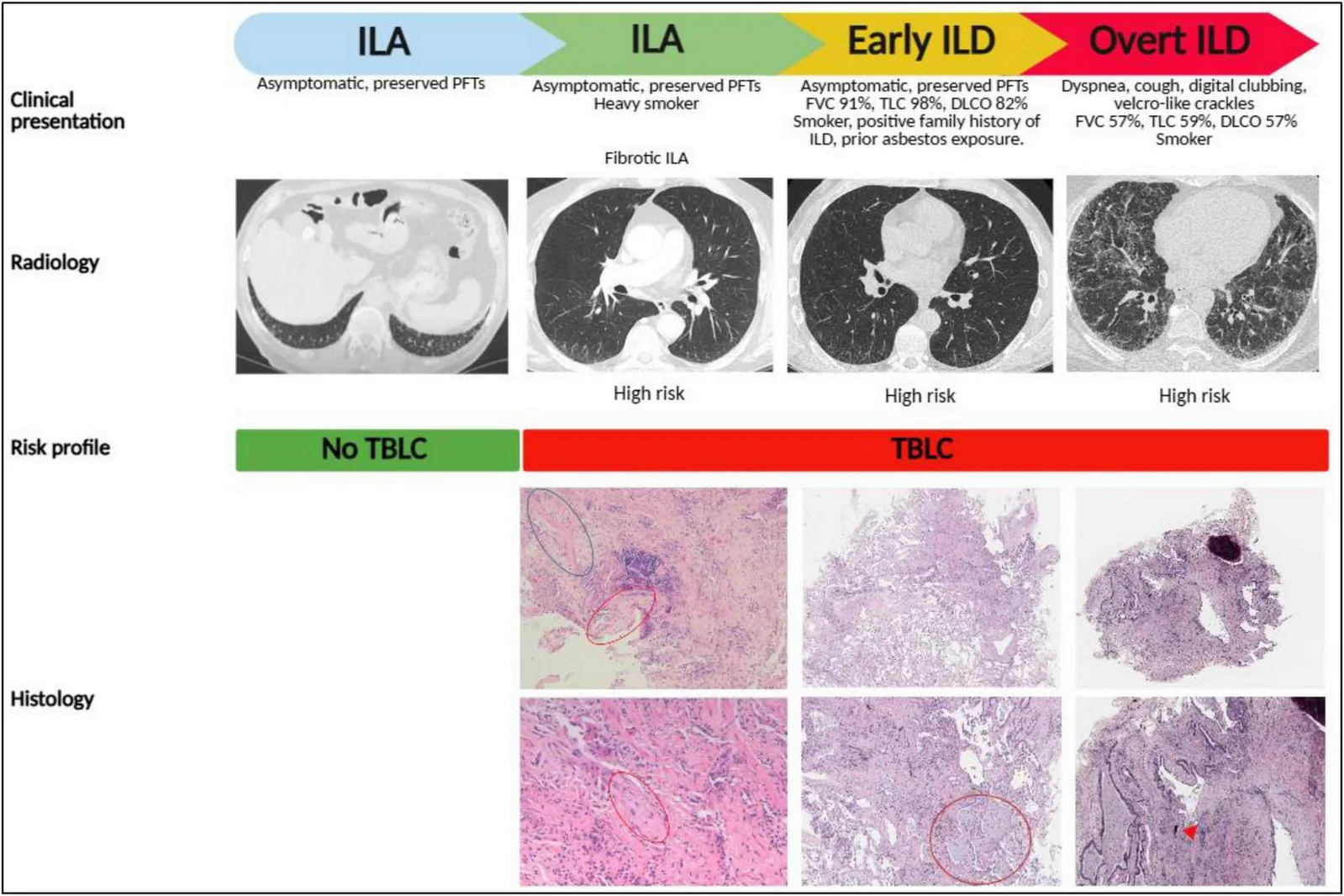
This figure illustrates the proposed continuum from ILA to overt fibrotic disease across patients spanning the risk spectrum, with entity definitions based on the two referenced works (85, 152). It highlights clinical scenarios ranging from low-risk incidental ILAs to higher-risk phenotypes where TBLC is considered. The progression emphasizes the shift from asymptomatic cases to those requiring invasive sampling within a multidisciplinary context.

Within this conceptual framework, Figure 2 illustrates the proposed continuum from ILA to early ILD and overt fibrotic disease, highlighting representative clinical scenarios across the spectrum of risk, from low-risk incidental ILAs not warranting invasive sampling, to progressively higher-risk phenotypes in which TBLC may be considered within a structured multidisciplinary context.

Ultimately, the field now faces a major paradigm shift: from observing early interstitial abnormalities as incidental radiological findings, to interrogating them as biologically active substrates of disease. Whether early histological characterization can meaningfully alter this trajectory remains unresolved, but it is precisely this uncertainty that justifies rigorous prospective investigation and defines the next frontier of fibrotic lung disease research.
